# Some Theoretical Foundations of Bare-Simulation Optimization of Some Directed Distances between Fuzzy Sets Respectively Basic Belief Assignments

**DOI:** 10.3390/e26040312

**Published:** 2024-04-01

**Authors:** Michel Broniatowski, Wolfgang Stummer

**Affiliations:** 1 Laboratoire de Probabilités, Statistique et Modélisation, Sorbonne Université, 4 Place Jussieu, 75252 Paris, France; michel.broniatowski@sorbonne-universite.fr; 2 Department of Mathematics, Friedrich-Alexander-Universität Erlangen–Nürnberg, Cauerstrasse 11, 91058 Erlangen, Germany

**Keywords:** generalized *φ*–divergences, fuzzy sets, basic belief assignments

## Abstract

It is well known that in information theory—as well as in the adjacent fields of statistics, machine learning and artificial intelligence—it is essential to quantify the dissimilarity between objects of uncertain/imprecise/inexact/vague information; correspondingly, constrained optimization is of great importance, too. In view of this, we define the dissimilarity-measure-natured *generalized φ–divergences* between fuzzy sets, ν–rung orthopair fuzzy sets, extended representation type ν–rung orthopair fuzzy sets as well as between those fuzzy set types and vectors. For those, we present how to tackle corresponding constrained minimization problems by appropriately applying our recently developed dimension-free *bare (pure) simulation method*. An analogous program is carried out by defining and optimizing *generalized φ–divergences* between (rescaled) basic belief assignments as well as between (rescaled) basic belief assignments and vectors.

## 1. Introduction

Directed distances—particularly known as divergences—D(Q,P) between probability vectors (i.e., vectors of probability frequencies) Q,P are widely used in statistics as well as in the adjacent research fields of information theory, artificial intelligence and machine learning. Prominent examples are, e.g., the Kullback–Leibler information distance/divergence (also known as relative entropy), the Hellinger distance, the Jensen–Shannon divergence and Pearson’s chi-square distance/divergence; those are special cases of the often-used wider class of the so-called Csiszár–Ali–Silvey–Morimoto (CASM) φ–divergences $D_{\varphi }\left(Q,P\right)$ (cf. [1,2,3]). For some comprehensive overviews on CASM φ–divergences, the reader is referred to, e.g., the insightful books [4,5,6,7,8,9], the survey articles [10,11,12,13], and the references therein. It is well known that the optimization of such CASM φ–divergences plays an important role, in obtaining estimators (e.g., the omnipresent maximum likelihood estimation method can be equivalently seen as a minimum Kullback–Leibler information distance estimation method), as well as in quantifying the model adequacy in the course of a model-search (model-selection) procedure; for the latter, see, e.g., [14,15,16,17].

In the literature, one can also find a substantial amount of special cases of CASM φ–divergences $D_{\varphi }\left(Q,P\right)$ between other prominent statistical quantities Q, P (other than probability frequencies), see, e.g., [18] for a corresponding recent survey. In contrast, there also exist special cases of CASM φ–divergences $D_{\varphi }\left(B,A\right)$ between other basic objects *B*,*A* for the quantification of uncertain/imprecise/inexact/vague information such as *fuzzy sets* (cf. [19]) and *basic belief assignments* from Dempster–Shafer evidence theory (cf. [20,21]). Indeed, as far as the former is concerned, for instance, ref. [22] employ (a variant of) the Kullback–Leibler information distance between two fuzzy sets *B* and *A* (which they call *fuzzy expected information for discrimination in favor of B against A*), ref. [23] investigate the Jensen–Shannon divergence between two intuitionistic fuzzy sets *B* and *A* (which they call *symmetric information measure between B and A*), whereas [24] deal with the Jensen–Shannon divergence between two extended representation type (i.e., hesitancy-degree including) Pythagorean fuzzy sets *B* and *A*. As far as CASM φ–divergences $D_{\varphi }\left(B,A\right)$ between basic belief assignments (BBAs) *B*, *A* is concerned, for instance, refs. [25,26] employ the Jensen–Shannon divergence for multi-sensor data fusion, whereas [27] use the Hellinger distance for characterizing the degree of conflict between BBAs.

In view of the above-mentioned illuminations, the main goals of this paper are as follows:

(M1) to define—dissimilarity-quantifying— *generalized CASM φ–divergences* between fuzzy sets, between ν–rung orthopair fuzzy sets in the sense of [28] (including intuitionistic and Pythagorean fuzzy sets), between extended representation type ν–rung orthopair fuzzy sets, between those fuzzy set types and vectors, between (rescaled) basic belief assignments as well as between (rescaled) basic belief assignments and vectors;
(M2) to present how one can tackle corresponding constrained minimization problems by appropriately applying our recently developed dimension-free *bare (pure) simulation method* of [29].

This agenda is achieved in the following way: in the next Section 2, we recall the basic definitions and properties of *generalized CASM φ–divergences* between vectors. The follow-up Section 3 explains the basic principles of our above-mentioned bare-simulation optimization method of [29] (where, for the sake of brevity, we focus on the minimal values and not on the corresponding minimizers). In Section 4, we achieve the main goals (M1) and (M2) for the above-mentioned types of fuzzy sets, whereas Section 5 concerns with (M1) and (M2) for (rescaled) basic belief assignments. The conclusions are discussed in the final Section 6.

## 2. Divergences between Vectors

As usual, a divergence is a function $D:R^{K}\times R^{K}\mapsto \left[0,\infty \right]$ with the following properties: D(Q,P)≥0 for *K*–dimensional vectors $Q,P\in R^{K}$, and D(Q,P)=0 iff Q=P. Since, in general, D(Q,P)≠D(P,Q) and the triangle inequality is not satisfied, D(Q,P) can be interpreted as *directed distance* from Q to P; accordingly, the divergences *D* can be connected to dissimilarity quantification and to geometric issues in various different ways, see, e.g., the detailed discussion in Section 1.5 of [18]. Typically, a divergence *D* is generated by some function φ. For the latter, here we fundamentally require the following:

(G1) φ:]−∞,∞[→[0,∞] is lower semicontinuous and convex;
(G2) φ(1)=0;
(G3) the effective domain dom(φ):={t∈R:φ(t)<∞} has interior int(dom(φ)) of the form int(dom(φ))=]a,b[ for some −∞≤a<1<b≤∞ (notice that (G3) follows from (G1), (G2) and the requirement that int(dom(φ)) is non-empty);
(G4’) φ is strictly convex in a neighborhood $]t_{-}^{sc},t_{+}^{sc}[\;\subseteq \;]a,b[$ of one ($t_{-}^{sc}<1<t_{+}^{sc}$).

Furthermore, we set $\varphi \left(a\right):=\lim_{t↓a}\varphi \left(t\right)$ and $\varphi \left(b\right):=\lim_{t↑b}\varphi \left(t\right)$. The class of all functions φ with (G1), (G2), (G3) and (G4’) will be denoted by $\tilde{\Upsilon }\left(\right]a,b\left[\right)$. For $\varphi \in \tilde{\Upsilon }\left(]a,b[\right)$, $P:=\left(p_{1},\ldots ,p_{K}\right)\in R_{>0}^{K}:=\left\{R:=\left(r_{1},\ldots ,r_{K}\right)\in R^{K}:\;r_{i}>0\;\mathrm{for}\;\mathrm{all}\;i=1,\ldots ,K\right\}$ and $Q:=\left(q_{1},\ldots ,q_{K}\right)\in \Omega \subset R^{K}$ we define as directed distance the *generalized Csiszár–Ali–Silvey–Morimoto φ–divergence* (cf. [1,2,3,30,31]) —in short, the *generalized φ–divergence*—

(1) $$D_{\varphi }\left(Q,P\right)\;:=\;\sum_{k=1}^{K}p_{k}\cdot \varphi \left(\frac{q_{k}}{p_{k}}\right)\;\geq 0,$$

where for finiteness reasons one often even has $Q\in \Omega \subset R_{\geq 0}^{K}:=\left\{R:=\left(r_{1},\ldots ,r_{K}\right)\in R^{K}:\;r_{i}\geq 0\;\mathrm{for}\;\mathrm{all}\;i=1,\ldots ,K\right\}$; for a comprehensive technical treatment, see, e.g., [32] (for instance, if ]a,b[=]0,∞[ then one can include zeros in (1) by the “conventions” $p_{k}\cdot \varphi \left(\frac{0}{p_{k}}\right)=p_{k}\cdot \lim_{t↓0}\varphi \left(t\right)$ for $p_{k}>0$, $0\cdot \varphi \left(\frac{q_{k}}{0}\right)=q_{k}\cdot \frac{0}{q_{k}}\cdot \varphi \left(\frac{q_{k}}{0}\right)=q_{k}\cdot \lim_{t↓0}t\cdot \varphi \left(\frac{1}{t}\right)$ for $q_{k}>0$, and $0\cdot \varphi \left(\frac{0}{0}\right)=0$). Comprehensive overviews on these important (generalized) φ–divergences are given in e.g., [4,5,6,7,8,9,10,11,12,13,14,15,16,17,18], and the references therein.

## 3. Optimization of Generalized φ–Divergences via the Bare Simulation Solution Method

In the following we deal with

**Problem** **1.** *For pregiven $\varphi \in \tilde{\Upsilon }\left(\right]a,b\left[\right)$, positive-entries vector $P:=\left(p_{1},\ldots ,p_{K}\right)\in R_{>0}^{K}$ (or from some subset thereof), and subset $\Omega \subset R^{K}$ with regularity properties*

(2) $$cl\left(\Omega \right)=cl\left(int\left(\Omega \right)\right),\;int\left(\Omega \right)\neq \emptyset ,$$

*find*

(3) $$\underset{Q\in \Omega }{\inf}D_{\varphi }\left(Q,P\right),$$

*provided that*

(4) $$\underset{Q\in \Omega }{\inf}D_{\varphi }\left(Q,P\right)<\infty .$$

**Remark** **1.** (a) When ***Ω*** is not closed but merely satisfies *(2)*, then the infimum in *(3)* may not be reached in ***Ω*** although it is finite; additionally, ***Ω*** is a closed set, then a minimizer $Q^{*}\in \Omega$ exists. In the subsetup where ***Ω*** is a closed convex set and int(Ω)≠∅, *(2)* is satisfied and the minimizer $Q^{*}\in \Omega$ in *(3)* is attained and even unique. When ***Ω*** is open and satisfies *(2)*, then the infimum in *(3) *exists, but is generally reached at some generalizedprojection of P on ***Ω*** (see [33] for the Kullback–Leibler divergence case of probability measures, which extends to any generalized φ–divergence in our framework). However, in this paper, we only deal with finding the infimum/minimum in *(3)* (rather than a corresponding minimizer).

*(b) Our approach is predestined for non- or semiparametric models. For instance, *(2)* is valid for the appropriate tubular neighborhoods of parametric models or for more general non-parametric settings, such as, e.g., shape constraints.*

*(c) Without further mentioning, the regularity condition *(2)* is supposed to hold in the full topology.*

According to our work [29], the above-mentioned Problem 1 can be solved by a new dimension-free precise *bare simulation (BS) method* to be explained in the following. We first suppose

**Condition** **1.** *With $M_{P}:=\sum_{i=1}^{K}p_{i}>0$, the divergence generator φ in *(3)* is such that its multiple $\tilde{\varphi }:=M_{P}\cdot \varphi$ satisfies (G1) to (G4’) (i.e., $\tilde{\varphi }\in \tilde{\Upsilon }\left(\right]a,b\left[\right)$) and additionally there holds the representation*

(5) $$\tilde{\varphi }\left(t\right)=\underset{z\in R}{\sup}\left(z\cdot t-\log\int_{R}e^{z\cdot y}\;d\tilde{\zeta \;⃓}\left(y\right)\right),\;t\in R,$$

*for some probability measure $\tilde{\zeta \;⃓}$ on the real line R such that the function $z\mapsto \int_{R}e^{z\cdot y}d\tilde{\zeta \;⃓}$ *(*y*)* is finite on some open interval containing zero (notice that the latter implies that $\int_{R}y\;d$ $\tilde{\zeta \;⃓}$ *(*y*)* *=* *1* and that $\tilde{\zeta \;⃓}$ has light tails).*

A detailed discussion on the representability (5) can be found in [34]. By means of this *cornerstone Condition* 1, we construct in [29] a sequence ${\left(\xi_{n}^{\tilde{W}}\right)}_{n\in N}$ of $R^{K}$–valued random variables/random vectors (on an auxiliary probability space (X,A,ℿ)) as follows: for any n∈N and any $k\in \left\{1,\ldots ,K\right\}$, let $n_{k}:=\left\lfloor n\cdot {\tilde{p}}_{k}\right\rfloor$ where ⌊x⌋ denotes the integer part of *x* and ${\tilde{p}}_{k}:=p_{k}/M_{P}>0$. Thus, one has $\lim_{n\to \infty }\frac{n_{k}}{n}={\tilde{p}}_{k}.$ Moreover, we assume that n∈N is large enough, namely, $n\geq \max_{k\in \{1,\ldots ,K\}}\frac{1}{{\tilde{p}}_{k}}$, and decompose the set {1,…,n} of all integers from 1 to *n* into the following disjoint blocks: $I_{1}^{\left(n\right)}:=\left\{1,\ldots ,n_{1}\right\}$, $I_{2}^{\left(n\right)}:=\left\{n_{1}+1,\ldots ,n_{1}+n_{2}\right\}$, and so on until the last block $I_{K}^{\left(n\right)}:=\left\{\sum_{k=1}^{K-1}n_{k}+1,\ldots ,n\right\}$, which, therefore, contains all integers from $n_{1}+\ldots +n_{K-1}+1$ to *n*. Clearly, $I_{k}^{\left(n\right)}$ has $n_{k}\geq 1$ elements (i.e., $\mathrm{card}\left(I_{k}^{\left(n\right)}\right)=n_{k}$, where card(A) denotes the number of elements in a set *A*) for all k∈{1,…,K−1}, and the last block $I_{K}^{\left(n\right)}$ has $n-\sum_{k=1}^{K-1}n_{k}\geq 1$ elements, which, anyhow, satisfies $\lim_{n\to \infty }\mathrm{card}\left(I_{K}^{\left(n\right)}\right)/n={\tilde{p}}_{K}$. Furthermore, consider a vector $\tilde{W}:=\left({\tilde{W}}_{1},\ldots ,{\tilde{W}}_{n}\right)$ where the ${\tilde{W}}_{i}$’s are i.i.d. copies of the random variable $\tilde{W}$ whose distribution is associated with the multiple divergence-generator $\tilde{\varphi }$ through (5), in the sense that $ℿ\left[\tilde{W}\in \cdot \;\right]=\;\zeta \;⃓\;$ [·]. We group the ${\tilde{W}}_{i}$’s according to the above-mentioned blocks and sum them up blockwise, in order to build the following *K*–component random vector

(6) $$\xi_{n}^{\tilde{W}}:=\left(\frac{1}{n}\sum_{i\in I_{1}^{\left(n\right)}}{\tilde{W}}_{i},\ldots ,\frac{1}{n}\sum_{i\in I_{K}^{\left(n\right)}}{\tilde{W}}_{i}\right).$$

For such a context, in [29], we obtain the following solution of Problem 1:

**Theorem** **1.** *Under Condition 1, there holds the “bare-simulation (BS) minimizability”*

$$\underset{Q\in \Omega }{\inf}D_{\varphi }\left(Q,P\right)=-\lim_{n\to \infty }\frac{1}{n}\log ℿ\left[\xi_{n}^{\tilde{W}}\in \Omega \right]$$

*for any $\Omega \subset R^{K}$ with regularity properties *(2)* and finiteness property *(4)*.*

Theorem 1 provides our principle for the *approximation* of the solution of the deterministic optimization problem (3). Indeed, by replacing the involved limit by its finite counterpart, we deduce for given large *n*

(7) $$\underset{Q\in \Omega }{\inf}D_{\varphi }\left(Q,P\right)\;\approx \;-\frac{1}{n}\log ℿ\left[\xi_{n}^{\tilde{W}}\in \Omega \right];$$

it remains to estimate the probability on the right-hand side of (7). The latter can be performed either by a *naive estimator* of the frequency of those replications of $\xi_{n}^{\tilde{W}}$ which hit Ω, or, more efficiently, by some improved estimator, see [29] for details, where we give concrete construction methods as well as numerous solved cases; for the latter, for the sake of brevity, we mention only two important special cases. The first one deals with the class of power-divergence generators $\varphi :=\tilde{c}\cdot \varphi_{\gamma }:R\mapsto \left[0,\infty \right]$ (with arbitrary multiplier $\tilde{c}\in \;]0,\infty [$) defined by

(8) $$\begin{array}{ccc} \tilde{c}\cdot \varphi_{\gamma }\left(t\right) & := & \left\{\begin{array}{cc} \tilde{c}\cdot \frac{t^{\gamma }-\gamma \cdot t+\gamma -1}{\gamma \cdot (\gamma -1)}, & \mathrm{if}\gamma \in \;]-\infty ,0\left[\;\mathrm{and}t\in \right]0,\infty [,\\ \tilde{c}\cdot \left(-\log t+t-1\right), & \mathrm{if}\gamma =0\;\mathrm{and}t\in ]0,\infty [,\\ \tilde{c}\cdot \frac{t^{\gamma }-\gamma \cdot t+\gamma -1}{\gamma \cdot (\gamma -1)}, & \mathrm{if}\gamma \in \;]0,1[\;\mathrm{and}t\in [0,\infty [,\\ \tilde{c}\cdot \left(t\cdot \log t+1-t\right), & \mathrm{if}\gamma =1\;\mathrm{and}t\in [0,\infty [,\\ \tilde{c}\cdot \frac{{\left(t-1\right)}^{2}}{2}, & \mathrm{if}\gamma =2\;\mathrm{and}t\in \;]-\infty ,\infty [,\\ \tilde{c}\cdot \left\{\frac{t^{\gamma }-\gamma \cdot t+\gamma -1}{\gamma \cdot (\gamma -1)}\cdot 1_{]0,\infty [}\left(t\right)+\left(\frac{1}{\gamma }-\frac{t}{\gamma -1}\right)\cdot 1_{]-\infty ,0]}\left(t\right)\right\}, & \mathrm{if}\gamma \in \;]2,\infty \left[\;\mathrm{and}t\in \;\right]-\infty ,\infty [,\\ \infty , & \mathrm{else}, \end{array}\right. \end{array}$$

which—by (1)—generate (the vector-valued form of) the *generalized power divergences* given by

(9) $$\begin{array}{ccc} & & \;D_{\tilde{c}\cdot \varphi_{\gamma }}\left(Q,P\right)\\ & & \;:=\left\{\begin{array}{cc} \tilde{c}\cdot \left\{\frac{\sum_{k=1}^{K}{\left(q_{k}\right)}^{\gamma }\cdot {\left(p_{k}\right)}^{1-\gamma }}{\gamma \cdot (\gamma -1)}-\frac{1}{\gamma -1}\cdot \sum_{k=1}^{K}q_{k}+\frac{1}{\gamma }\cdot \sum_{k=1}^{K}p_{k}\right\}, & \mathrm{if}\gamma \in \;]-\infty ,0[,\;P\in R_{>0}^{K}\;\mathrm{and}Q\in R_{>0}^{K},\\ \tilde{c}\cdot \left\{\sum_{k=1}^{K}p_{k}\cdot \log\left(\frac{p_{k}}{q_{k}}\right)+\sum_{k=1}^{K}q_{k}-\sum_{k=1}^{K}p_{k}\right\}, & \mathrm{if}\gamma =0,\;P\in R_{>0}^{K}\;\mathrm{and}Q\in R_{>0}^{K},\\ \tilde{c}\cdot \left\{\frac{\sum_{k=1}^{K}{\left(q_{k}\right)}^{\gamma }\cdot {\left(p_{k}\right)}^{1-\gamma }}{\gamma \cdot (\gamma -1)}-\frac{1}{\gamma -1}\cdot \sum_{k=1}^{K}q_{k}+\frac{1}{\gamma }\cdot \sum_{k=1}^{K}p_{k}\right\}, & \mathrm{if}\gamma \in \;]0,1[,\;P\in R_{>0}^{K}\;\mathrm{and}Q\in R_{\geq 0}^{K},\\ \tilde{c}\cdot \left\{\sum_{k=1}^{K}q_{k}\cdot \log\left(\frac{q_{k}}{p_{k}}\right)-\sum_{k=1}^{K}q_{k}+\sum_{k=1}^{K}p_{k}\right\}, & \mathrm{if}\gamma =1,\;P\in R_{>0}^{K}\;\mathrm{and}Q\in R_{\geq 0}^{K},\\ \tilde{c}\cdot \sum_{k=1}^{K}\frac{{\left(q_{k}-p_{k}\right)}^{2}}{2\cdot p_{k}}, & \mathrm{if}\gamma =2,\;P\in R_{>0}^{K}\;\mathrm{and}Q\in R^{K},\\ \tilde{c}\cdot \left\{\sum_{k=1}^{K}\frac{{\left(q_{k}\right)}^{\gamma }\cdot {\left(p_{k}\right)}^{1-\gamma }}{\gamma \cdot (\gamma -1)}\cdot 1_{[0,\infty [}\left(q_{k}\right)-\frac{1}{\gamma -1}\cdot \sum_{k=1}^{K}q_{k}+\frac{1}{\gamma }\cdot \sum_{k=1}^{K}p_{k}\right\}, & \mathrm{if}\gamma \in \;]2,\infty [,\;P\in R_{>0}^{K}\;\mathrm{and}Q\in R^{K},\\ \infty , & \mathrm{else}; \end{array}\right. \end{array}$$

for a corresponding detailed literature embedding (including applications and transformations), the reader is referred to [29]. For any fixed $M_{P}:=\sum_{i=1}^{K}p_{i}\in \;]0,\infty [$, Condition 1 is satisfied for $\varphi :=\tilde{c}\cdot \varphi_{\gamma }$ for all $\tilde{c}\in \;]0,\infty [$ and all γ∈R∖]1,2[, and thus the BS-minimizability concerning Theorem 1 can be applied. Notice that the case γ∈]1,2[ has to be left out for technical reasons. The corresponding crucial simulation distributions $ℿ\left[\tilde{W}\in \cdot \;\right]=\;\zeta \;⃓\;$ [·] (cf. (5)) are given by the following:

(DIS1) a tilted stable distribution on [0,∞[ for the case γ∈]−∞,0[;
(DIS2) the “$Gamma(M_{P}\cdot \tilde{c},M_{P}\cdot \tilde{c})$ distribution” for γ=0;
(DIS3) the “$Compound\;Poisson(\frac{M_{P}\cdot \tilde{c}}{\gamma })$ – $Gamma(\frac{M_{P}\cdot \tilde{c}}{1-\gamma },\frac{\gamma }{1-\gamma })$ distribution” for γ∈]0,1[;
(DIS4) the “$\frac{1}{M_{P}\cdot \tilde{c}}$–fold of $Poisson(M_{P}\cdot \tilde{c})$ distribution” for γ=1;
(DIS5) the “$Normal(1,\frac{1}{M_{P}\cdot \tilde{c}})$ distribution” for γ=2;
(DIS6) a distorted stable distribution on ]−∞,∞[ for γ∈]2,∞[.

for details see our paper [29].

The second important special case to be mentioned here, deals with

(10) $$\begin{array}{ccc} \varphi_{snKL,\tilde{c}}\left(t\right)\; & := & \;\left\{\begin{array}{cc} \tilde{c}\cdot \left[\;t\cdot \log t+\left(t+1\right)\cdot \log\left(\frac{2}{t+1}\right)\;\right]\;\in \;[0,\infty [, & \mathrm{if}t\in \;]0,\infty [,\\ \tilde{c}\cdot \log2, & \mathrm{if}t=0,\\ \infty , & \mathrm{if}t\in \;]-\infty ,0[, \end{array}\right. \end{array}$$

which leads to

(11) $$D_{\varphi_{snKL,\tilde{c}}}\left(Q,P\right)=\tilde{c}\cdot \left\{\sum_{k=1}^{K}q_{k}\cdot \log\left(\frac{2q_{k}}{q_{k}+p_{k}}\right)+\sum_{k=1}^{K}p_{k}\cdot \log\left(\frac{2p_{k}}{q_{k}+p_{k}}\right)\right\},\;\;\;\mathrm{if}\;P\in R_{>0}^{K},Q\in R_{\geq 0}^{K}.$$

For any fixed $M_{P}:=\sum_{i=1}^{K}p_{i}\in \;]0,\infty [$, Condition 1 is satisfied for $\varphi :=\varphi_{snKL,\tilde{c}}$ for all $\tilde{c}\in \;]0,\infty [$, and thus the BS-minimizability concerning Theorem 1 can be applied. The corresponding crucial simulation distribution $ℿ\left[\tilde{W}\in \cdot \;\right]=\;\zeta \;⃓\;$ [·] (cf. (5)) is given by the “$\frac{1}{M_{P}\cdot \tilde{c}}$–fold of $NegativeBinomial(M_{P}\cdot \tilde{c},\frac{1}{2})$” (cf. [29]). For the special subcase $\tilde{c}=1$ we derive

(12) $$D_{\varphi_{snKL,1}}\left(Q,P\right)=D_{\varphi_{1}}\left(Q,\left(Q+P\right)/2\right)+D_{\varphi_{1}}\left(P,\left(Q+P\right)/2\right),\;\mathrm{if}\;P\in R_{>0}^{K},Q\in R_{\geq 0}^{K}\;\;\mathrm{and}\;\;\sum_{i=1}^{K}p_{i}=\sum_{i=1}^{K}q_{i},$$

which means that in such a situation the divergence (11) can be rewritten as a sum of two generalized Kullback–Leibler divergences (cf. (9)). For the important subsetup that $\sum_{i=1}^{K}p_{i}=\sum_{i=1}^{K}q_{i}=1$, and thus both P=ℙ as well as Q=ℚ are probability vectors, the divergence $D_{\varphi_{snKL,1}}\left(\mathbb{Q},\mathbb{P}\right)$ in (12) is the well-known (cf. [35,36,37,38,39,40,41]) *Jensen–Shannon divergence* (being also called symmetrized and normalized Kullback–Leibler divergence, symmetrized and normalized relative entropy, and capacitor discrimination).

For further examples the reader is referred to our paper [29]. In the latter, we also derive bare-simulation optimization versions for constraint sets Ω in—a strictly positive multiple A>0 of—the probability simplex, to be explained in the following. First, we denote by $S^{K}:=\left\{Q:=\left(q_{1},\ldots ,q_{K}\right)\in R_{\geq 0}^{K}:\;\sum_{i=1}^{K}q_{i}=1\right\}$ to be the simplex of probability vectors (probability simplex), and $S_{>0}^{K}:=\left\{Q:=\left(q_{1},\ldots ,q_{K}\right)\in R_{>0}^{K}:\;\sum_{i=1}^{K}q_{i}=1\right\}$. For better emphasis, (as already performed above) for elements of these two sets we use the symbols ℚ,ℙ instead of Q,P, etc., but for their components we still use our notation $q_{k}$,$p_{k}$. Moreover, subsets of $S^{K}$ or $S_{>0}^{K}$ will be denoted by ΩΩ instead of Ω, etc. As indicated above, in the following we deal with constraint sets of the form A·ΩΩ for some arbitrary A∈]0,∞[, which automatically satisfy $int\left(A\cdot \Omega \;\Omega \right)=\emptyset$ in the *full topology* and thus the regularity condition (2) is violated (cf. Remark 1 (c)). Therefore, we need an adaption of the above-mentioned method. In more detail, we deal with

**Problem** **2.** *For pregiven $\varphi \in \tilde{\Upsilon }\left(\right]a,b\left[\right)$, positive-components vector $P:=\left(p_{1},\ldots ,p_{K}\right)\in R_{>0}^{K}$, and subset $A\cdot \Omega \;\Omega \subset A\cdot S^{K}$ with regularity properties—in the relative topology (!!) —*

(13) $$cl\left(A\cdot \Omega \;\Omega \right)=cl\left(int\left(A\cdot \Omega \;\Omega \right)\right),\;int\left(A\cdot \Omega \;\Omega \right)\neq \emptyset ,$$

*find*

(14) $$\underset{Q\in A\cdot \Omega \;\Omega }{\inf}D_{\varphi }\left(Q,P\right),$$

*provided that*

$$\underset{Q\in A\cdot \Omega \;\Omega }{\inf}D_{\varphi }\left(Q,P\right)<\infty$$

*and that divergence generator φ additionally satisfies the Condition 1.*

For the directed distance minimization Problem 2, we proceed (with the same notations) as above and construct the following *K*–component random vector (instead of $\xi_{n}^{\tilde{W}}$ in (6))

(15) $$\begin{array}{ccc} \xi_{n}^{w\tilde{W}} & := & \left\{\begin{array}{c} \left(\frac{\sum_{i\in I_{1}^{\left(n\right)}}{\tilde{W}}_{i}}{\sum_{k=1}^{K}\sum_{i\in I_{k}^{\left(n\right)}}{\tilde{W}}_{i}},\ldots ,\frac{\sum_{i\in I_{K}^{\left(n\right)}}{\tilde{W}}_{i}}{\sum_{k=1}^{K}\sum_{i\in I_{k}^{\left(n\right)}}{\tilde{W}}_{i}}\right),\;\mathrm{if}\sum_{j=1}^{n}{\tilde{W}}_{j}\neq 0,\\ \;\left(\infty ,\ldots ,\infty \right)=:\infty ,\;\mathrm{if}\sum_{j=1}^{n}{\tilde{W}}_{j}=0. \end{array}\right. \end{array}$$

By construction, in case of $\sum_{j=1}^{n}{\tilde{W}}_{j}\neq 0$, the sum of the random *K* vector components of (15) are now automatically equal to one, but—as (depending on φ) the ${\tilde{W}}_{i}$’s may take both positive and negative values— these random components may be negative with a probability strictly greater than zero (respectively, non-negative with a probability strictly less than one). However, $ℿ[\xi_{n}^{w\tilde{W}}\in S_{>0}^{K}]>0$ since all the (identically distributed) random variables ${\tilde{W}}_{i}$ have and expectation of one (as a consequence of the assumed representability (5)); in case of $ℿ[{\tilde{W}}_{1}>0]=1$, one has even $ℿ[\xi_{n}^{w\tilde{W}}\in S_{>0}^{K}]=1$. Summing up things, the probability $ℿ[\xi_{n}^{w\tilde{W}}\in \Omega \;\Omega ]$ is strictly positive and finite at least for large n, whenever $\inf_{Q\in A\cdot \Omega \;\Omega }D_{\varphi }\left(Q,P\right)$ is finite.

As mentioned right after (9) above, the required representability (5) is satisfied for all (multiples of) the generators $\varphi \left(\cdot \right):=\tilde{c}\cdot \varphi_{\gamma }\left(\cdot \right)$ of (8) with $\tilde{c}\in \;]0,\infty [$ and γ∈R∖]1,2[ (cf. [29]). Within this context, for arbitrary constants $\overset{˘}{A}>0$ and $\overset{˘}{c}>0$ we define the auxiliary functions

$$\begin{array}{ccc} H_{\gamma ,\overset{˘}{c},\overset{˘}{A}}\left(z\right)\; & := & \;\left\{\begin{array}{c} \frac{\overset{˘}{c}}{\gamma \cdot (\gamma -1)}\cdot \left\{{\overset{˘}{A}}^{\gamma }\cdot {\left[1-\frac{\gamma }{\overset{˘}{c}}\cdot z\right]}^{-\left(\gamma -1\right)}-1-\gamma \cdot \left(\overset{˘}{A}-1\right)\right\},\;\mathrm{if}\gamma \in \;]-\infty ,0\left[\;\cup \;\right]0,1[\;\cup \;[2,\infty [\\ \;\mathrm{and}z\in R\mathrm{such}\mathrm{that}\gamma \cdot z\leq \overset{˘}{c},\;\\ z-\overset{˘}{c}\cdot \left(1-\overset{˘}{A}+\log\overset{˘}{A}\right),\;\mathrm{if}\gamma =0\;\mathrm{and}z\in R,\\ \overset{˘}{c}\cdot \left\{1-\overset{˘}{A}-\overset{˘}{A}\cdot \left[\log\left(1-\frac{z}{\overset{˘}{c}}\right)-\log\overset{˘}{A}\right]\right\},\;\mathrm{if}\gamma =1\;\mathrm{and}z\in \;]-\infty ,\overset{˘}{c}[, \end{array}\right. \end{array}$$

we obtained (with slight rescalation) in [29] the following solution of Problem 2:

**Theorem** **2.** *Let $P\in R_{>0}^{K}$ with $M_{P}:=\sum_{i=1}^{K}p_{i}$, $\tilde{c}\in \;]0,\infty [$, γ∈R∖]1,2[ and A∈]0,∞[ be arbitrary but fixed. Moreover, let $\overset{˘}{c}:=M_{P}\cdot \tilde{c}$, $\overset{˘}{A}:=\frac{A}{M_{P}}$ and ${\left({\tilde{W}}_{i}\right)}_{i\in N}$ be a family of independent and identically distributed R–valued random variables with probability distribution  ζ⃓  *[·]*:=$ℿ\left[{\tilde{W}}_{1}\in \cdot \;\right]$ being connected with the divergence generator $\varphi :=\tilde{c}\cdot \varphi_{\gamma }\left(\cdot \right)$ via the representability *(5)* (cf. (DIS1)-(DIS6)). Then there holds the “bare-simulation (BS) minimizability”*

(16) $$\begin{array}{c} \underset{Q\in A\cdot \;\Omega \;\Omega \;}{\inf}D_{\tilde{c}\cdot \varphi_{\gamma }}\left(Q,P\right)=H_{\gamma ,\overset{˘}{c},\overset{˘}{A}}\left(-\lim_{n\to \infty }\frac{1}{n}\log ℿ\left[\xi_{n}^{w\tilde{W}}\in \;\Omega \;\Omega \;\right]\right) \end{array}$$

*for all sets A·ΩΩ$\subset {\tilde{M}}_{\gamma }$ satisfying the regularity properties *(13)* in the relative topology. Here, ${\tilde{M}}_{\gamma }:=A\cdot S_{>0}^{K}$ for γ∈]−∞,0], respectively, ${\tilde{M}}_{\gamma }:=A\cdot S^{K}$ for γ∈]0,1]∪[2,∞[.*

Analogously to (7), Theorem 2 provides our principle for the *approximation* of the solution of the deterministic optimization problem (14). Indeed, by replacing the involved limit by its finite counterpart, we deduce for given large *n*

(17) $$\underset{Q\in A\cdot \;\Omega \;\Omega \;}{\inf}D_{\tilde{c}\cdot \varphi_{\gamma }}\left(Q,P\right)\;\approx \;H_{\gamma ,\overset{˘}{c},\overset{˘}{A}}\left(-\frac{1}{n}\log ℿ\left[\xi_{n}^{w\tilde{W}}\in \;\Omega \;\Omega \;\right]\right);$$

the probability in the latter can be estimated either by a *naive estimator* of the frequency of those replications of $\xi_{n}^{w\tilde{W}}$ which hit ΩΩ, or more efficiently by some improved estimator; see [29] for details, where (for the case A=1, with straightforward adaption to A≠1) we give concrete construction methods as well as numerous solved cases.

By means of the straightforward deterministic transformations, Theorem 2 carries straightforwardly over to the BS optimizability of, e.g., the following important quantities

(18) $$\begin{array}{ccc} \;R_{\gamma }\left(Q,P\right)\; & := & \;\frac{\log\left(\sum_{k=1}^{K}{\left(q_{k}\right)}^{\gamma }\cdot {\left(p_{k}\right)}^{1-\gamma }\right)}{\gamma \cdot (\gamma -1)}\\ \; & = & \;\frac{\log\left(\left(1-\gamma \right)\cdot M_{P}+\gamma \cdot A+\gamma \cdot \left(\gamma -1\right)\cdot D_{\varphi_{\gamma }}\left(Q,P\right)\right)}{\gamma \cdot (\gamma -1)},\\ & & \mathrm{if}\gamma \in \;]-\infty ,0[\;\cup \;]0,1[\;\cup \;[\;2,\infty [, \end{array}$$

(provided that all involved power divergences are finite), which are (by monotonicity and continuity) thus BS-minimizable on Ω=A·ΩΩ for all γ∈]−∞,0[∪]0,1[∪[2,∞[:

(19) $$\begin{array}{c} \underset{Q\in A\cdot \;\Omega \;\Omega \;}{\inf}R_{\gamma }\left(Q,P\right)=\lim_{n\to \infty }\frac{\log\left(\left(1-\gamma \right)\cdot M_{P}+\gamma \cdot A+\gamma \cdot \left(\gamma -1\right)\cdot H_{\gamma ,\overset{˘}{c},\overset{˘}{A}}\left(-\frac{1}{n}\log ℿ\left[\xi_{n}^{w\tilde{W}}\in \;\Omega \;\Omega \;\right]\right)\right)}{\gamma \cdot (\gamma -1)} \end{array}$$

for all sets A·ΩΩ$\subset {\tilde{M}}_{\gamma }$ satisfying the regularity properties (13) *in the relative topology*; here, the simulation distribution $ℿ\left[\tilde{W}\in \cdot \;\right]=\;\zeta \;⃓\;$ [·] (cf. (5)) is given by (DIS1), (DIS3), (DIS5) and (DIS6), respectively. The special subcase A=1, $M_{P}=1$ in (18) (and thus, Q, P are probability vectors ℚ,ℙ) corresponds to the prominent *Renyi divergences/distances* [42] (in the scaling of, e.g., [4] and in probability vector form), see, e.g., [43] for a comprehensive study of their properties. Notice that $R_{\gamma }\left(Q,P\right)$ may become strictly negative, e.g., in the case that $\left(1-\gamma \right)\cdot M_{P}+\gamma \cdot A\in \;]0,1[$, however, in this case the variant

(20) $$R_{\gamma }^{nn}\left(Q,P\right):=R_{\gamma }\left(Q,P\right)-\frac{log\left(\left(1-\gamma \right)\cdot M_{P}+\gamma \cdot A\right)}{\gamma \cdot (\gamma -1)}$$

always stays non-negative and leads basically to the “same” optimization

(21) $$\underset{Q\in A\cdot \Omega \;\Omega }{\inf}R_{\gamma }^{nn}\left(Q,P\right)=\left(\underset{Q\in A\cdot \Omega \;\Omega }{\inf}R_{\gamma }\left(Q,P\right)\right)-\frac{log\left(\left(1-\gamma \right)\cdot M_{P}+\gamma \cdot A\right)}{\gamma \cdot (\gamma -1)}\;.$$

For the cases γ=1 and γ=0, important transformations are the *modified Kullback–Leibler information (modified relative entropy)*

(22) $$\begin{array}{c} -M_{P}\;\leq \;I\left(Q,P\right):=\sum_{k=1}^{K}q_{k}\cdot \log\left(\frac{q_{k}}{p_{k}}\right)\;=\;D_{\varphi_{1}}\left(Q,P\right)+A-M_{P} \end{array}$$

and the *modified reverse Kullback–Leibler information (modified reverse relative entropy)*

$$\begin{array}{c} M_{P}-A\;\leq \;\tilde{I}\left(Q,P\right):=\sum_{k=1}^{K}p_{k}\cdot \log\left(\frac{p_{k}}{q_{k}}\right)\;=\;D_{\varphi_{0}}\left(Q,P\right)+M_{P}-A; \end{array}$$

notice that I(Q,P) can become negative if $A<M_{P}$ and $\tilde{I}\left(Q,P\right)$ can become negative if $A>M_{P}$ (see [30] for counterexamples). Nevertheless, in general, we obtain immediately from γ=1 in Theorem 2 that

(23) $$\begin{array}{cc} \; & \underset{Q\in A\cdot \;\Omega \;\Omega \;}{\inf}I\left(Q,P\right)=\lim_{n\to \infty }\left(A-M_{P}-\frac{1}{n}\log ℿ\left[\xi_{n}^{w\tilde{W}}\in \;\Omega \;\Omega \;\right]\right) \end{array}$$

for all sets A·ΩΩ$\subset {\tilde{M}}_{1}$ satisfying the regularity properties (13) *in the relative topology*; here, the simulation distribution $ℿ\left[\tilde{W}\in \cdot \;\right]=\;\zeta \;⃓\;$ [·] (cf. (5)) is given by (DIS4). Moreover, by employing γ=0 in Theorem 2 we deduce

(24) $$\begin{array}{cc} \; & \underset{Q\in A\cdot \;\Omega \;\Omega \;}{\inf}\tilde{I}\left(Q,P\right)=\lim_{n\to \infty }\left(M_{P}-A-\frac{1}{n}\log ℿ\left[\xi_{n}^{w\tilde{W}}\in \;\Omega \;\Omega \;\right]\right) \end{array}$$

for all sets A·ΩΩ$\subset {\tilde{M}}_{0}$ satisfying the regularity properties (13) *in the relative topology*; here, the simulation distribution $ℿ\left[\tilde{W}\in \cdot \;\right]=\;\zeta \;⃓\;$ [·] (cf. (5)) is given by (DIS2).

**Remark** **2.** *By taking the special case ***P***:=${\mathbb{P}}^{unif}:=\left(\frac{1}{K},\ldots ,\frac{1}{K}\right)$ to be the probability vector of frequencies of the uniform distribution on {1,…,K} (and thus $M_{P}=1$) in the above formulas *(16)* to *(24)*, we can deduce many bare-simulation solutions of constrained optimization of various different versions of entropies Q↦E(Q); for details, see Sections VIII and XII in [29].*

From (19), (21), (23) and (24) we deduce the approximations (for large n∈N)

(25) $$\underset{Q\in A\cdot \;\Omega \;\Omega \;}{\inf}R_{\gamma }\left(Q,P\right)\;\approx \;\frac{\log\left(\left(1-\gamma \right)\cdot M_{P}+\gamma \cdot A+\gamma \cdot \left(\gamma -1\right)\cdot H_{\gamma ,\overset{˘}{c},\overset{˘}{A}}\left(-\frac{1}{n}\log ℿ\left[\xi_{n}^{w\tilde{W}}\in \;\Omega \;\Omega \;\right]\right)\right)}{\gamma \cdot (\gamma -1)},$$

(26) $$\begin{array}{ccc} \underset{Q\in A\cdot \;\Omega \;\Omega \;}{\inf}R_{\gamma }^{nn}\left(Q,P\right) & \approx & \frac{\log\left(\left(1-\gamma \right)\cdot M_{P}+\gamma \cdot A+\gamma \cdot \left(\gamma -1\right)\cdot H_{\gamma ,\overset{˘}{c},\overset{˘}{A}}\left(-\frac{1}{n}\log ℿ\left[\xi_{n}^{w\tilde{W}}\in \;\Omega \;\Omega \;\right]\right)\right)}{\gamma \cdot (\gamma -1)}\\ & & -\;\frac{log\left(\left(1-\gamma \right)\cdot M_{P}+\gamma \cdot A\right)}{\gamma \cdot (\gamma -1)}, \end{array}$$

(27) $$\underset{Q\in A\cdot \;\Omega \;\Omega \;}{\inf}I\left(Q,P\right)\;\approx \;\left(A-M_{P}-\frac{1}{n}\log ℿ\left[\xi_{n}^{w\tilde{W}}\in \;\Omega \;\Omega \;\right]\right),$$

(28) $$\underset{Q\in A\cdot \;\Omega \;\Omega \;}{\inf}\tilde{I}\left(Q,P\right)\;\approx \;\left(M_{P}-A-\frac{1}{n}\log ℿ\left[\xi_{n}^{w\tilde{W}}\in \;\Omega \;\Omega \;\right]\right),$$

where for the involved $ℿ\left[\xi_{n}^{w\tilde{W}}\in \Omega \;\Omega \right]$ one can again employ either a *naive estimator* of the frequency of those replications of $\xi_{n}^{w\tilde{W}}$ which hit ΩΩ, or an improved estimator, see [29] for details.

## 4. Minimization Problems with Fuzzy Sets

Our above-mentioned BS framework can be applied to the—imprecise/inexact/vague information describing — *fuzzy sets* (cf. [19]) and optimization problems on divergences between those. Indeed, let $Y=\left\{d_{1},\ldots ,d_{K}\right\}$ be a finite set (called the *universe (of discourse)*), C⊂Y and $M^{C}:Y\mapsto \left[0,1\right]$ be a corresponding *membership function*, where $M^{C}\left(d_{k}\right)$ represents the degree/grade of membership of the element $d_{k}$ to the set *C*; accordingly, the object $C^{*}:=\left\{\langle x,M^{C}\left(x\right)\rangle |x\in Y\right\}$ is called a *fuzzy set in Y* (or fuzzy subset of Y). Moreover, if A⊂Y and B⊂Y are two unequal sets, then the corresponding membership functions $M^{A}$ and $M^{B}$ should be unequal. Furthermore, we model the *vector of membership degrees to C* by $P^{C}:={\left(p_{k}^{C}\right)}_{k=1,\ldots ,K}:={\left(M^{C}\left(d_{k}\right)\right)}_{k=1,\ldots ,K}$, which satisfies the *key constraint* $0\leq p_{k}^{C}\leq 1$ for all k∈{1,…,K} and, consequently, the *aggregated key constraint* $0\leq \sum_{k=1}^{K}p_{k}^{C}\leq K$ (as a side remark, $\sum_{k=1}^{K}M^{C}\left(d_{k}\right)$ is called power of the fuzzy set $C^{*}$). For divergence generators φ in $\tilde{\Upsilon }\left(\right]a,b\left[\right)$ with (say) 0≤a<1<b and for two sets A,B⊂Y we can apply (1) to the corresponding membership functions and define the generalized φ–divergence $D_{\varphi }\left(B^{*},A^{*}\right)$ between the fuzzy sets $B^{*}$ and $A^{*}$ (on the same universe Y) as (cf. [44])

(29) $$D_{\varphi }\left(B^{*},A^{*}\right):=D_{\varphi }\left(P^{B},P^{A}\right)=\sum_{k=1}^{K}p_{k}^{A}\cdot \varphi \left(\frac{p_{k}^{B}}{p_{k}^{A}}\right)=\sum_{k=1}^{K}M^{A}\left(d_{k}\right)\cdot \varphi \left(\frac{M^{B}\left(d_{k}\right)}{M^{A}\left(d_{k}\right)}\right)\;\geq 0$$

(depending on φ, zero degree values may have to be excluded for finiteness). For instance, we can take $\varphi \left(t\right):=\varphi_{1}\left(t\right):=t\cdot \log t+1-t\in [0,\infty [$ for t∈[0,∞[ (cf. (8)) to end up with a generalized Kullback–Leibler divergence (generalized relative entropy) between $B^{*}$ and $A^{*}$ given by (cf. (9))

$$\begin{array}{ccc} & & \;D_{\tilde{c}\cdot \varphi_{1}}\left(B^{*},A^{*}\right)=D_{\tilde{c}\cdot \varphi_{1}}\left(P^{B},P^{A}\right)=\tilde{c}\cdot \left\{\sum_{k=1}^{K}M^{B}\left(d_{k}\right)\cdot \log\left(\frac{M^{B}\left(d_{k}\right)}{M^{A}\left(d_{k}\right)}\right)-\sum_{k=1}^{K}M^{B}\left(d_{k}\right)+\sum_{k=1}^{K}M^{A}\left(d_{k}\right)\right\}\;\geq \;0,\\ & & \;\mathrm{if}M^{A}\left(d_{k}\right)>0\;\mathrm{and}M^{B}\left(d_{k}\right)\geq 0\;\mathrm{for}\mathrm{all}k=1,\ldots ,K; \end{array}$$

this contrasts the choice $\varphi \left(t\right):=\overset{˘}{\varphi }\left(t\right):=t\cdot \log t\in [-\frac{1}{e},\infty [$ of [22]

$$\begin{array}{ccc} & & \;D_{\overset{˘}{\varphi }}\left(B^{*},A^{*}\right)=I\left(P^{B},P^{A}\right)=\tilde{c}\cdot \left\{\sum_{k=1}^{K}M^{B}\left(d_{k}\right)\cdot \log\left(\frac{M^{B}\left(d_{k}\right)}{M^{A}\left(d_{k}\right)}\right)\right\},\\ & & \;\mathrm{if}M^{A}\left(d_{k}\right)>0\;\mathrm{and}M^{B}\left(d_{k}\right)\geq 0\;\mathrm{for}\mathrm{all}k=1,\ldots ,K, \end{array}$$

which they call *fuzzy expected information for discrimination in favor of B against A*, and which *may become negative* (cf. see the discussion after (22) and [30] in a more general context). Returning to the general case (29), as a special case of the above-mentioned BS concepts, we can tackle optimization problems of the type

$$\underset{B^{*}\in \Omega^{*}}{\inf}D_{\varphi }\left(B^{*},A^{*}\right):=\underset{P^{B}\in \Omega }{\inf}D_{\varphi }\left(P^{B},P^{A}\right)$$

where $\Omega^{*}$ is a collection of fuzzy sets (on the same universe Y) whose membership degree vectors form the set Ω satisfying (2) and (4). Because of the inequality type key constraint

$$0\leq M^{B}\left(d_{k}\right)\leq 1\;\mathrm{for}\mathrm{all}k\in \{1,\ldots ,K\}$$

which is incorporated into Ω, and which implies that Ω is contained in the *K*–dimensional unit hypercube and in particular $0\leq \sum_{k=1}^{K}p_{k}^{B}\leq K$, Theorem 1 (and thus, (7)) applies correspondingly (e.g., to the generalized power divergences $D_{\tilde{c}\cdot \varphi_{\gamma }}\left(B^{*},A^{*}\right)=D_{\tilde{c}\cdot \varphi_{\gamma }}\left(P^{B},P^{A}\right)$ given by (9) and the generalized Jensen–Shannon divergence $D_{\varphi_{snKL,\tilde{c}}}\left(B^{*},A^{*}\right)=D_{\varphi_{snKL,\tilde{c}}}\left(P^{B},P^{A}\right)$ given by (11))—unless there is a more restrictive constraint that violates (2) such as, e.g., $\sum_{k=1}^{K}p_{k}^{B}=c$ with c≤K for which Theorem 2 (and hence, (17))—as well as its consequences (19), (21), (23), (24) (and thus, (25)–(28)) can be employed.

The above-mentioned considerations can be extended to the recent concept of *ν–rung orthopair fuzzy sets* (cf. [28]) and divergences between those. Indeed, for C⊂Y, besides a membership function $M^{C}:Y\mapsto \left[0,1\right]$ one additionally models a *nonmembership function* $N^{C}:Y\mapsto \left[0,1\right]$, where $N^{C}\left(d_{k}\right)$ represents the degree/grade of nonmembership of the element $d_{k}$ to the set *C*. Moreover, if A⊂Y and B⊂Y are unequal sets, then the corresponding nonmembership functions $N^{A}$ and $N^{B}$ should be unequal. For fixed ν∈[1,∞[, the *key constraint*

(30) $$0\leq {\left(M^{C}\left(d_{k}\right)\right)}^{\nu }+{\left(N^{C}\left(d_{k}\right)\right)}^{\nu }\leq 1\;\mathrm{for}\mathrm{all}k\in \{1,\ldots ,K\}$$

is required to be satisfied, too. Accordingly, the object $C^{**}:=\left\{\langle x,M^{C}\left(x\right),N^{C}\left(x\right)\rangle |x\in Y\right\}$ is called a *ν–rung orthopair fuzzy set in Y (or … subset of Y)*. The object $C^{**}$ is called *intuitionistic fuzzy set in Y* (cf. [45]) in case of ν=1, and *Pythagorean fuzzy set in Y* (cf. [46,47]) in the case of ν=2. For the choice ν=1 together with $N^{C}\left(x\right):=1-M^{C}\left(x\right)$, the object $C^{**}$ can be regarded as an extended representation of the fuzzy set $C^{*}$ in Y.

For any ν–rung orthopair fuzzy set $C^{**}$ in Y, we model the corresponding *vector of concatenated membership and nonmembership degrees to C* by $P^{C}:={\left(p_{k}^{C}\right)}_{k=1,\ldots ,2K}:=\left(M^{C}\left(d_{1}\right),\ldots M^{C}\left(d_{K}\right),N^{C}\left(d_{1}\right),\ldots N^{C}\left(d_{K}\right)\right)$, which, due to (30), satisfies the *aggregated key constraint*

(31) $$0\leq \sum_{k=1}^{2K}{\left(p_{k}^{C}\right)}^{\nu }\leq K;$$

in other words, $P^{C}$ lies (within the 2K–dimensional Euclidean space) in the intersection of the first/positive orthant with the ν–norm ball centered at the origin and with radius $K^{1/\nu }$. Analogously to (29), we can define the generalized φ–divergence $D_{\varphi }\left(B^{**},A^{**}\right)$ between the ν–rung orthopair fuzzy sets $B^{**}$ and $A^{**}$ (on the same universe Y) as (cf. [44])

(32) $$\begin{array}{ccc} & & D_{\varphi }\left(B^{**},A^{**}\right):=D_{\varphi }\left(P^{B},P^{A}\right)=\sum_{k=1}^{2K}p_{k}^{A}\cdot \varphi \left(\frac{p_{k}^{B}}{p_{k}^{A}}\right)\\ & & =\sum_{k=1}^{K}\left\{M^{A}\left(d_{k}\right)\cdot \varphi \left(\frac{M^{B}\left(d_{k}\right)}{M^{A}\left(d_{k}\right)}\right)+N^{A}\left(d_{k}\right)\cdot \varphi \left(\frac{N^{B}\left(d_{k}\right)}{N^{A}\left(d_{k}\right)}\right)\right\}\;\geq 0 \end{array}$$

respectively, as its variant (cf. [44])

(33) $$\begin{array}{ccc} & & \;D_{\varphi }^{var}\left(B^{**},A^{**}\right):=D_{\varphi }\left({\left(P^{B}\right)}^{\nu },{\left(P^{A}\right)}^{\nu }\right):=\sum_{k=1}^{2K}{\left(p_{k}^{A}\right)}^{\nu }\cdot \varphi \left(\frac{{\left(p_{k}^{B}\right)}^{\nu }}{{\left(p_{k}^{A}\right)}^{\nu }}\right)\\ & & \;=\sum_{k=1}^{K}\left\{{\left(M^{A}\left(d_{k}\right)\right)}^{\nu }\cdot \varphi \left(\frac{{\left(M^{B}\left(d_{k}\right)\right)}^{\nu }}{{\left(M^{A}\left(d_{k}\right)\right)}^{\nu }}\right)+{\left(N^{A}\left(d_{k}\right)\right)}^{\nu }\cdot \varphi \left(\frac{{\left(N^{B}\left(d_{k}\right)\right)}^{\nu }}{{\left(N^{A}\left(d_{k}\right)\right)}^{\nu }}\right)\right\}\;\geq 0. \end{array}$$

For the special choice $\varphi \left(t\right):=\varphi_{snKL,1}\left(t\right)$ (cf. (10)) and ν=1, the definition (32) leads to the generalized Jensen–Shannon divergence between $B^{**}$ and $A^{**}$ given by

$$\begin{array}{ccc} & & D_{\varphi_{snKL,1}}\left(B^{**},A^{**}\right):=D_{\varphi_{snKL,1}}\left(P^{B},P^{A}\right)\\ & & =\sum_{k=1}^{K}\{M^{B}\left(d_{k}\right)\cdot \log\left(\frac{2M^{B}\left(d_{k}\right)}{M^{B}\left(d_{k}\right)+M^{A}\left(d_{k}\right)}\right)+M^{A}\left(d_{k}\right)\cdot \log\left(\frac{2M^{A}\left(d_{k}\right)}{M^{B}\left(d_{k}\right)+M^{A}\left(d_{k}\right)}\right)\\ & & +N^{B}\left(d_{k}\right)\cdot \log\left(\frac{2N^{B}\left(d_{k}\right)}{N^{B}\left(d_{k}\right)+N^{A}\left(d_{k}\right)}\right)+N^{A}\left(d_{k}\right)\cdot \log\left(\frac{2N^{A}\left(d_{k}\right)}{N^{B}\left(d_{k}\right)+N^{A}\left(d_{k}\right)}\right)\}\;\geq 0,\\ & & \;\mathrm{if}\;M^{A}\left(d_{k}\right)>0,N^{A}\left(d_{k}\right)>0,M^{B}\left(d_{k}\right)\geq 0,N^{B}\left(d_{k}\right)\geq 0,\;\;\mathrm{for}\mathrm{all}k\in \{1,\ldots ,K\}; \end{array}$$

this coincides with the symmetric information measure between $B^{**}$ and $A^{**}$ of [23]. For the special choice $\varphi \left(t\right):=\varphi_{1}\left(t\right):=t\cdot \log t+1-t\in [0,\infty [$ with t∈[0,∞[ (cf. (8)), ν=1, $N^{A}\left(x\right):=1-M^{A}\left(x\right)$, $N^{B}\left(x\right):=1-M^{B}\left(x\right)$ and thus (31) turning into $\sum_{k=1}^{2K}{\left(p_{k}^{A}\right)}^{\nu }=\sum_{k=1}^{2K}{\left(p_{k}^{B}\right)}^{\nu }=K$, one can straightforwardly see that the outcoming generalized Kullback–Leibler divergence (generalized relative entropy) between $B^{**}$ and $A^{**}$ given by

$$\begin{array}{ccc} & & \;0\leq D_{\varphi_{1}}\left(B^{**},A^{**}\right)=D_{\varphi_{1}}\left(P^{B},P^{A}\right)\\ & & \;=\sum_{k=1}^{K}M^{B}\left(d_{k}\right)\cdot \log\left(\frac{M^{B}\left(d_{k}\right)}{M^{A}\left(d_{k}\right)}\right)-\sum_{k=1}^{K}M^{B}\left(d_{k}\right)+\sum_{k=1}^{K}M^{A}\left(d_{k}\right)+\sum_{k=1}^{K}N^{B}\left(d_{k}\right)\cdot \log\left(\frac{N^{B}\left(d_{k}\right)}{N^{A}\left(d_{k}\right)}\right)-\sum_{k=1}^{K}N^{B}\left(d_{k}\right)+\sum_{k=1}^{K}N^{A}\left(d_{k}\right)\\ & & \;=\sum_{k=1}^{K}M^{B}\left(d_{k}\right)\cdot \log\left(\frac{M^{B}\left(d_{k}\right)}{M^{A}\left(d_{k}\right)}\right)+\sum_{k=1}^{K}N^{B}\left(d_{k}\right)\cdot \log\left(\frac{N^{B}\left(d_{k}\right)}{N^{A}\left(d_{k}\right)}\right),\\ & & \;\mathrm{if}M^{A}\left(d_{k}\right)>0,N^{A}\left(d_{k}\right)>0\;\mathrm{and}M^{B}\left(d_{k}\right)\geq 0,N^{B}\left(d_{k}\right)\geq 0\;\mathrm{for}\mathrm{all}k=1,\ldots ,K, \end{array}$$

coincides with $D_{\overset{˘}{\varphi }}\left(B^{**},A^{**}\right)$ where $\overset{˘}{\varphi }\left(t\right):=t\cdot \log t$; the latter divergence was used, e.g., in [22] under the name *average fuzzy information for discrimination in favor of B against A*.

Returning to the general context, in terms of the divergences (32) and (33), we can tackle—as a special case of the above-mentioned BS concepts—optimization problems of the type

$$\begin{array}{ccc} & & \underset{B^{**}\in \Omega^{**}}{\inf}D_{\varphi }\left(B^{**},A^{**}\right):=\underset{P^{B}\in \Omega }{\inf}D_{\varphi }\left(P^{B},P^{A}\right)\;\mathrm{respectively}\\ & & \underset{B^{**}\in \Omega^{**}}{\inf}D_{\varphi }^{var}\left(B^{**},A^{**}\right):=\underset{P^{B}\in \Omega }{\inf}D_{\varphi }\left({\left(P^{B}\right)}^{\nu },{\left(P^{A}\right)}^{\nu }\right), \end{array}$$

where $\Omega^{**}$ is a collection of ν–rung orthopair fuzzy sets whose concatenated membership–nonmembership degree vectors form the set Ω satisfying (2) and (4) as well as (30) for C:=B. Because of the latter, Theorem 1 (and thus, (7)) applies correspondingly (e.g., to the generalized power divergences $D_{\tilde{c}\cdot \varphi_{\gamma }}\left(B^{**},A^{**}\right)=D_{\tilde{c}\cdot \varphi_{\gamma }}\left(P^{B},P^{A}\right)$ given by (9) and the generalized Jensen–Shannon divergence $D_{\varphi_{snKL,\tilde{c}}}\left(B^{**},A^{**}\right)=D_{\varphi_{snKL,\tilde{c}}}\left(P^{B},P^{A}\right)$ given by (11))—unless there is a more restrictive constraint that violates (2) such as, e.g., $\sum_{k=1}^{2K}p_{k}^{B}=c$ with c≤K for which Theorem 2 (and thus, (17)) — as well as its consequences (19), (21), (23), (24) (and thus, (25)–(28)) can be employed; such a situation appears, e.g., in the case ν=1 together with $N^{B}\left(x\right):=1-M^{B}\left(x\right)$, which leads to c=K.

For the ν–rung orthopair fuzzy sets $C^{**}$ in Y, we can also further “flexibilize” our divergences by additionally incorporating the *hesitancy degree* of the element $d_{k}$ to *C*, which is defined as

$$H^{C}\left(d_{k}\right):={\left(1-{\left(M^{C}\left(d_{k}\right)\right)}^{\nu }-{\left(N^{C}\left(d_{k}\right)\right)}^{\nu }\right)}^{1/\nu }\in \left[0,1\right]$$

(cf. [28]), and which implies the *key constraint*

(34) $${\left(H^{C}\left(d_{k}\right)\right)}^{\nu }+{\left(M^{C}\left(d_{k}\right)\right)}^{\nu }+{\left(N^{C}\left(d_{k}\right)\right)}^{\nu }=1\;\mathrm{for}\mathrm{all}k\in \{1,\ldots ,K\}.$$

Accordingly, the object $C^{***}:=\left\{\langle x,M^{C}\left(x\right),N^{C}\left(x\right),H^{C}\left(x\right)\rangle |x\in Y\right\}$ can be regarded as an extended representation of the ν–rung orthopair fuzzy set $C^{**}$ in Y. For $C^{***}$, we model the corresponding *vector of concatenated membership, nonmembership and hesitancy degrees to C* by

$$P^{C}:={\left(p_{k}^{C}\right)}_{k=1,\ldots ,3K}:=\left(M^{C}\left(d_{1}\right),\ldots M^{C}\left(d_{K}\right),N^{C}\left(d_{1}\right),\ldots N^{C}\left(d_{K}\right),H^{C}\left(d_{1}\right),\ldots H^{C}\left(d_{K}\right)\right)$$

which, due to (34), satisfies the *aggregated key constraint*

$$\sum_{k=1}^{3K}{\left(p_{k}^{C}\right)}^{\nu }=K;$$

in other words, $P^{C}$ lies (within the 3K–dimensional Euclidean space) in the intersection of the first/positive orthant with the ν–norm sphere centered at the origin and with radius $K^{1/\nu }$. Analogously to (32) and (33), we can define the generalized φ–divergence $D_{\varphi }\left(B^{***},A^{***}\right)$ between the extended representation type ν–rung orthopair fuzzy sets $B^{***}$ and $A^{***}$ (on the same universe Y) as (cf. [44])

(35) $$\begin{array}{ccc} & & D_{\varphi }\left(B^{***},A^{***}\right):=D_{\varphi }\left({\left(P^{B}\right)}^{\nu },{\left(P^{A}\right)}^{\nu }\right):=\sum_{k=1}^{3K}{\left(p_{k}^{A}\right)}^{\nu }\cdot \varphi \left(\frac{{\left(p_{k}^{B}\right)}^{\nu }}{{\left(p_{k}^{A}\right)}^{\nu }}\right)\\ & & =\sum_{k=1}^{K}\{{\left(M^{A}\left(d_{k}\right)\right)}^{\nu }\cdot \varphi \left(\frac{{\left(M^{B}\left(d_{k}\right)\right)}^{\nu }}{{\left(M^{A}\left(d_{k}\right)\right)}^{\nu }}\right)+{\left(N^{A}\left(d_{k}\right)\right)}^{\nu }\cdot \varphi \left(\frac{{\left(N^{B}\left(d_{k}\right)\right)}^{\nu }}{{\left(N^{A}\left(d_{k}\right)\right)}^{\nu }}\right)\\ & & \;+{\left(H^{A}\left(d_{k}\right)\right)}^{\nu }\cdot \varphi \left(\frac{{\left(H^{B}\left(d_{k}\right)\right)}^{\nu }}{{\left(H^{A}\left(d_{k}\right)\right)}^{\nu }}\right)\}\;\geq 0; \end{array}$$

for technical reasons, we do not deal with its variant

$$\begin{array}{ccc} & & D_{\varphi }^{var}\left(B^{***},A^{***}\right):=D_{\varphi }\left(P^{B},P^{A}\right)=\sum_{k=1}^{3K}p_{k}^{A}\cdot \varphi \left(\frac{p_{k}^{B}}{p_{k}^{A}}\right)\\ & & =\sum_{k=1}^{K}\left\{M^{A}\left(d_{k}\right)\cdot \varphi \left(\frac{M^{B}\left(d_{k}\right)}{M^{A}\left(d_{k}\right)}\right)+N^{A}\left(d_{k}\right)\cdot \varphi \left(\frac{N^{B}\left(d_{k}\right)}{N^{A}\left(d_{k}\right)}\right)+H^{A}\left(d_{k}\right)\cdot \varphi \left(\frac{H^{B}\left(d_{k}\right)}{H^{A}\left(d_{k}\right)}\right)\right\}\;\geq 0. \end{array}$$

For instance, by taking the special choice ν=2 and $\varphi \left(t\right):=\varphi_{snKL,1}\left(t\right)$ (cf. (10)) in (35), we arrive at the Jensen–Shannon divergence between $B^{***}$ and $A^{***}$ of the form $D_{\varphi_{snKL,1}}\left(B^{***},A^{***}\right):=D_{\varphi_{snKL,1}}\left(P^{B},P^{A}\right)$, which—by the virtue of (35) and (11) — coincides with the (squared) Pythagorean fuzzy set Jensen–Shannon divergence measure between $B^{***}$ and $A^{***}$ of [24].

To continue with the general context, as a particular application of the above-mentioned BS concepts, we can tackle the general optimization problems of the generalized power divergence type

(36) $$\begin{array}{ccc} & & \underset{B^{***}\in \Omega^{***}}{\inf}D_{\tilde{c}\cdot \varphi_{\gamma }}\left(B^{***},A^{***}\right):=\underset{P^{B}\in \Omega }{\inf}D_{\tilde{c}\cdot \varphi_{\gamma }}\left({\left(P^{B}\right)}^{\nu },{\left(P^{A}\right)}^{\nu }\right),\;\gamma \in R\setminus ]1,2\left[\;,\;\;\tilde{c}\in \;\right]0,\infty [\;, \end{array}$$

where $\Omega^{***}$ is a collection of extended representation type ν–rung orthopair fuzzy sets whose concatenated membership–nonmembership-hesitancy degree vectors form the set Ω, satisfying (34) for C:=B (for each member) as well as the regularity properties (13) *in the relative topology*. Thus, for the minimization of (36) we can apply our Theorem 2 (and, consequently, (17)) by choosing there (with a slight abuse of notation) $A=M_{P}=K$. Of course, we can also apply our BS optimization method to the corresponding Renyi divergences $R_{\gamma }\left(B^{***},A^{***}\right):=R_{\gamma }\left({\left(P^{B}\right)}^{\nu },{\left(P^{A}\right)}^{\nu }\right)$ (via (19)), $R_{\gamma }^{nn}\left(B^{***},A^{***}\right):=R_{\gamma }^{nn}\left({\left(P^{B}\right)}^{\nu },{\left(P^{A}\right)}^{\nu }\right)$ (via (19) and (21)) as well as $I\left(B^{***},A^{***}\right):=I\left({\left(P^{B}\right)}^{\nu },{\left(P^{A}\right)}^{\nu }\right)$ (via (23)), $\tilde{I}\left(B^{***},A^{***}\right):=\tilde{I}\left({\left(P^{B}\right)}^{\nu },{\left(P^{A}\right)}^{\nu }\right)$ (via (24)), and employ the correspondingly applied approximations (25)–(28). For instance, by applying (20) with $A=M_{P}=K$ (with a slight abuse of notation) we arrive at the non-negative *γ–order Renyi divergence between ν–rung orthopair fuzzy sets* given by

(37) $$\begin{array}{ccc} & & \;0\leq R_{\gamma }^{nn}\left(B^{***},A^{***}\right):=R_{\gamma }^{nn}\left({\left(P^{B}\right)}^{\nu },{\left(P^{A}\right)}^{\nu }\right):=\frac{1}{\gamma \cdot (\gamma -1)}\cdot \left[\log\left(\sum_{k=1}^{3K}{\left({\left(p_{k}^{B}\right)}^{\nu }\right)}^{\gamma }\cdot {\left({\left(p_{k}^{A}\right)}^{\nu }\right)}^{1-\gamma }\right)-\log\left(K\right)\right]\\ & & \;=\frac{1}{\gamma \cdot (\gamma -1)}\cdot [\log(\sum_{k=1}^{K}\{{\left({\left(M^{B}\left(d_{k}\right)\right)}^{\nu }\right)}^{\gamma }\cdot {\left({\left(M^{A}\left(d_{k}\right)\right)}^{\nu }\right)}^{1-\gamma }+{\left({\left(N^{B}\left(d_{k}\right)\right)}^{\nu }\right)}^{\gamma }\cdot {\left({\left(N^{A}\left(d_{k}\right)\right)}^{\nu }\right)}^{1-\gamma }\\ & & \;+{\left({\left(H^{B}\left(d_{k}\right)\right)}^{\nu }\right)}^{\gamma }\cdot {\left({\left(H^{A}\left(d_{k}\right)\right)}^{\nu }\right)}^{1-\gamma }\})-\log\left(K\right)]\;\geq 0;\;\gamma \in \;]-\infty ,0\left[\;\cup \;\right]0,1[\;\cup \;[\;2,\infty [; \end{array}$$

depending on γ, zero degree values may have to be excluded for finiteness. As a side remark, let us mention that our divergence (37) contrasts to the recent (first) divergence of [48] who basically uses a different scaling, the product $\prod_{k=1}^{K}$ instead of the sum $\sum_{k=1}^{K}$, as well as $\frac{{\left(p_{k}^{A}\right)}^{\nu }+{\left(p_{k}^{B}\right)}^{\nu }}{2}$ instead of ${\left(p_{k}^{A}\right)}^{\nu }$. By appropriately applying (19) and (21), we can tackle with our BS method for γ∈]−∞,0[∪]0,1[∪[2,∞[ the minimization problem $\inf_{B^{***}\in \Omega^{***}}R_{\gamma }^{nn}\left(B^{***},A^{***}\right)$, where $\Omega^{***}$ is a collection of extended representation type ν–rung orthopair fuzzy sets $B^{***}$ whose concatenated membership–nonmembership-hesitancy degree vectors form the set Ω satisfying (34) for C:=B (for each member) as well as the regularity properties (13) *in the relative topology*.

We can also apply our BS optimization method to “crossover cases” $D_{\varphi }\left(B^{*},P\right):=D_{\varphi }\left(P^{B},P\right)$ and $D_{P,\varphi }\left(A^{*}\right):=D_{\varphi }\left(P,P^{A}\right)$ (instead of (29), $D_{\varphi }\left(B^{**},P\right):=D_{\varphi }\left(P^{B},P\right)$ and $D_{\varphi }\left(P,A^{**}\right):=D_{\varphi }\left(P,P^{A}\right)$ (instead of (32)), $D_{\varphi }^{var}\left(B^{**},P\right):=D_{\varphi }\left({\left(P^{B}\right)}^{\nu },P\right)$ and $D_{\varphi }^{var}\left(P,A^{**}\right):=D_{\varphi }\left(P,{\left(P^{A}\right)}^{\nu }\right)$ (instead of (33)), $D_{\varphi }\left(B^{***},P\right):=D_{\varphi }\left({\left(P^{B}\right)}^{\nu },P\right)$ and $D_{\varphi }\left(P,A^{***}\right):=D_{\varphi }\left(P,{\left(P^{A}\right)}^{\nu }\right)$ (instead of (35)), $R_{\gamma }\left(B^{***},P\right):=R_{\gamma }\left({\left(P^{B}\right)}^{\nu },P\right)$ and $R_{\gamma }\left(P,A^{***}\right):=R_{\gamma }\left(P,{\left(P^{A}\right)}^{\nu }\right)$, $R_{\gamma }^{nn}\left(B^{***},P\right):=R_{\gamma }^{nn}\left({\left(P^{B}\right)}^{\nu },P\right)$ and $R_{\gamma }^{nn}\left(P,A^{***}\right):=R_{\gamma }^{nn}\left(P,{\left(P^{A}\right)}^{\nu }\right)$, $I\left(B^{***},P\right):=I\left({\left(P^{B}\right)}^{\nu },P\right)$ and $I\left(P,A^{***}\right):=I\left(P,{\left(P^{A}\right)}^{\nu }\right)$, $\tilde{I}\left(B^{***},P\right):=\tilde{I}\left({\left(P^{B}\right)}^{\nu },P\right)$ and $\tilde{I}\left(P,A^{***}\right):=\tilde{I}\left(P,{\left(P^{A}\right)}^{\nu }\right)$, $D_{\tilde{c}\cdot \varphi_{\gamma }}\left(B^{***},P\right):=D_{\tilde{c}\cdot \varphi_{\gamma }}\left({\left(P^{B}\right)}^{\nu },P\right)$ and $D_{\tilde{c}\cdot \varphi_{\gamma }}\left(P,A^{***}\right):=D_{\tilde{c}\cdot \varphi_{\gamma }}\left(P,{\left(P^{A}\right)}^{\nu }\right)$, where $P\in R^{dim}$ (respectively, $R_{\geq 0}^{dim}$, $R_{>0}^{dim}$, $A\cdot S_{\geq 0}^{dim}$ or $A\cdot S_{>0}^{dim}$) is a general vector (not necessarily induced by a fuzzy set) having the same dimension dim (namely, *K*, 2K or 3K) as the fuzzy set induced vector to be compared with. For instance, if we apply Remark 2 to **P**:=${\mathbb{P}}^{unif}:=\left(\frac{1}{3K},\ldots ,\frac{1}{3K}\right)$ and $Q:={\left(P^{B}\right)}^{\nu }$ and employ the corresponding straightforward application of the general results of Sections VIII and XII of [29], then we end up (via the appropriately applied Theorem 2) with the BS optimization results of $D_{\tilde{c}\cdot \varphi_{\gamma }}(B^{***},{\mathbb{P}}^{unif}$), $R_{\gamma }(B^{***},{\mathbb{P}}^{unif}$), $I(B^{***},{\mathbb{P}}^{unif}$) and $\tilde{I}(B^{***},{\mathbb{P}}^{unif}$), which can be deterministically transformed into the BS optimization results of various different versions of entropies $E\left(B^{***}\right):=E\left({\left(P^{B}\right)}^{\nu }\right)$ of ν–rung orthopairs fuzzy sets $B^{***}$, where E(·) is any entropy in Chapter VIII of [29].

As a final remark of this section, let us mention that we can carry over the above-mentioned definitions and optimizations to (classical, intuitionistic, Pythagorean and ν–rung orthopair) *L*–fuzzy sets, where the range of the membership functions, nonmembership functions and hesitancy functions is an appropriately chosen lattice *L* (rather than L=[0,1]); for the sake of brevity, the details are omitted here.

## 5. Minimization Problems with Basic Belief Assignments

Our BS framework also covers—imprecise/inexact/vague information describing—*basic belief assignments* from Dempster–Shafer evidence theory (cf. [20,21]) and optimization problems on the divergences between those. Indeed, let $Y=\left\{d_{1},\ldots ,d_{K}\right\}$ be a finite set (called the *frame of discernment*) of mutually exclusive and collectively exhaustive events $d_{k}$. The corresponding power set of Y is denoted by $2^{Y}$ and has $2^{K}$ elements; we enumerate this by $2^{Y}:=\left\{A_{1},\ldots ,A_{2^{K}}\right\}$, where for convenience we set $A_{1}:=\emptyset$. A mapping $M:2^{Y}\mapsto \left[0,1\right]$ is called a *basic belief assignment (BBA)* (sometimes alternatively called basic probability assignment (BPA)) if it satisfies the two conditions

(38) $$M\left(\emptyset \right)=0\;\mathrm{and}\;\sum_{A\in 2^{Y}}M\left(A\right)\;=\;1.$$

Here, the belief mass M(A) reflects, e.g., the trust degree of evidence to proposition $A\in 2^{Y}$. From this, one can build the belief function $Bel:2^{Y}\mapsto \left[0,1\right]$ by $Bel\left(A\right):=\sum_{B:B\subseteq A}M\left(B\right)$ and the plausibility function $Pl:2^{Y}\mapsto \left[0,1\right]$ by $Pl\left(A\right):=\sum_{B:B\cap A\neq \emptyset }M\left(B\right)$. Moreover, we model the $2^{K}$–dimensional *vector of (M–based) BBA values* (vector of (*M*–based) belief masses) by $P^{M}:={\left(p_{k}^{M}\right)}_{k=1,\ldots ,2^{K}}:={\left(M(A_{k})\right)}_{k=1,\ldots ,2^{K}}$, which satisfies the *key constraint* $0\leq p_{k}^{M}\leq 1$ for all $k\in \{1,\ldots ,2^{K}\}$ and, by virtue of (38), the *aggregated key constraint* $\sum_{k=1}^{2^{K}}p_{k}^{M}=1$. Hence, $P^{M}$ lies formally in the $2^{K}$–dimensional simplex $S^{2^{K}}$ (but generally not in the corresponding probability vector-describing $S^{K}$).

For divergence generators φ in $\tilde{\Upsilon }\left(\right]a,b\left[\right)$ with (say) 0≤a<1<b and for two BBAs $M_{1},M_{2}$ on the same frame of discernment Y, we can apply (1) to the corresponding vectors of BBA values and define the *generalized φ–divergence $D_{\varphi }\left(M_{2},M_{1}\right)$ between the BBAs $M_{2}$ and $M_{1}$* (in short, *Belief–φ–divergence*) as

(39) $$D_{\varphi }\left(M_{2},M_{1}\right):=D_{\varphi }\left(P^{M_{2}},P^{M_{1}}\right)=\sum_{k=1}^{2^{K}}p_{k}^{M_{1}}\cdot \varphi \left(\frac{p_{k}^{M_{2}}}{p_{k}^{M_{1}}}\right)=\sum_{k=1}^{2^{K}}M_{1}\left(A_{k}\right)\cdot \varphi \left(\frac{M_{2}\left(A_{k}\right)}{M_{1}\left(A_{k}\right)}\right)\;\geq 0.$$

This definition (39) of the Belief–φ–divergence was first given in our paper [44]; later on, [49] used formally the same definition under the different (but deficient) assumptions that φ is only convex and satisfies φ(1)=0, which leads to a violation of the basic divergence properties (take, e.g., φ(t)=0 for all *t*, which implies $D_{\varphi }\left(P^{M_{2}},P^{M_{1}}\right)=0$ even if $P^{M_{2}}\neq P^{M_{1}}$; such an effect is excluded in our setup due to our assumption (G4’) being part of $\tilde{\Upsilon }\left(\right]a,b\left[\right)$); as a technical remark let us mention that (as already indicated in Section 2) depending on φ, zero belief masses may have to be excluded for finiteness in (39). For instance, we can take in (39) the special case $\varphi \left(t\right):=\varphi_{snKL,1}\left(t\right)$ (cf. (10)) to end up with the *Belief-Jensen–Shannon divergence* of [25,26] who applies this to multi-sensor data fusion. As another special case we can take $\varphi \left(t\right):=\varphi_{1/2}\left(t\right)$ (cf. (8)) to end up with the 4–times square of the *Hellinger distance of BBAs* of [27], who use this for characterizing the degree of conflict between BBAs. To continue with the general context, as a particular application of the above-mentioned BS concepts, we can tackle general optimization problems of the type

(40) $$\begin{array}{ccc} & & \underset{M_{2}\in \Omega^{BBA}}{\inf}D_{\tilde{c}\cdot \varphi_{\gamma }}\left(M_{2},M_{1}\right):=\underset{P^{M_{2}}\in \Omega }{\inf}D_{\tilde{c}\cdot \varphi_{\gamma }}\left(P^{M_{2}},P^{M_{1}}\right)\;\gamma \in R\setminus ]1,2\left[\;,\;\;\tilde{c}\in \;\right]0,\infty [\;, \end{array}$$

where $\Omega^{BBA}$ is a collection of BBAs whose vectors of BBA-values form the set $\Omega \in S^{2^{K}}$ satisfying the regularity properties (13) *in the relative topology*; hence, we can apply our Theorem 2 (and thus, (17)) for the minimization of (40), by taking $2^{K}$ instead of *K* as well as (with a slight abuse of notation) $A=M_{P^{M_{1}}}=1$. Of course, we can also apply our BS optimization method to the corresponding Renyi divergences $R_{\gamma }\left(M_{2},M_{1}\right):=R_{\gamma }\left(P^{M_{2}},P^{M_{1}}\right)$ (via (19)), $R_{\gamma }^{nn}\left(M_{2},M_{1}\right):=R_{\gamma }^{nn}\left(P^{M_{2}},P^{M_{1}}\right)$ (via (19) and (21)) as well as $I\left(M_{2},M_{1}\right):=I\left(P^{M_{2}},P^{M_{1}}\right)$ (via (23)), $\tilde{I}\left(M_{2},M_{1}\right):=\tilde{I}\left(P^{M_{2}},P^{M_{1}}\right)$ (via (24)), and employ the correspondingly applied approximations (25)–(28). For instance, by applying (18) and (19) we arrive at

$$\begin{array}{ccc} \;R_{\gamma }\left(M_{2},M_{1}\right):=R_{\gamma }\left(P^{M_{2}},P^{M_{1}}\right)\; & = & \;\frac{\log\left(\sum_{k=1}^{2^{K}}{\left(M_{2}\left(A_{k}\right)\right)}^{\gamma }\cdot {\left(M_{1}\left(A_{k}\right)\right)}^{1-\gamma }\right)}{\gamma \cdot (\gamma -1)}\;,\\ & & \mathrm{if}\gamma \in \;]-\infty ,0[\;\cup \;]0,1[\;\cup \;[\;2,\infty [, \end{array}$$

and

$$\begin{array}{cc} \; & \underset{M_{2}\in \Omega^{BBA}}{\inf}R_{\gamma }\left(M_{2},M_{1}\right)=\underset{P^{M_{2}}\in \Omega }{\inf}R_{\gamma }\left(P^{M_{2}},P^{M_{1}}\right)=\lim_{n\to \infty }\frac{\log\left(1+\gamma \cdot \left(\gamma -1\right)\cdot H_{\gamma ,\overset{˘}{c},\overset{˘}{A}}\left(-\frac{1}{n}\log\;\;\left[\xi_{n}^{w\tilde{W}}\in \Omega \right]\right)\right)}{\gamma \cdot (\gamma -1)} \end{array}$$

where $\xi_{n}^{w\tilde{W}}$ is chosen according to Theorem 2 with $2^{K}$ instead of *K* as well as $A=M_{P^{M_{1}}}=1$.

We can also apply our BS optimization method to the divergences between the rescaling of BBAs. For instance, let $\overset{˘}{M}\left(A\right):=\frac{M(A)}{2^{\left|A\right|}-1}$ ($A\in 2^{Y}$) with the convention that $\frac{0}{0}:=0$, and denote the corresponding vector $P^{\overset{˘}{M}}:={\left(p_{k}^{\overset{˘}{M}}\right)}_{k=1,\ldots ,2^{K}}:={\left(\overset{˘}{M}\left(A_{k}\right)\right)}_{k=1,\ldots ,2^{K}}$. Accordingly, we define the *generalized φ–divergence $D_{\varphi }\left({\overset{˘}{M}}_{2},{\overset{˘}{M}}_{1}\right)$ between the rescaled BBAs ${\overset{˘}{M}}_{2}$ and ${\overset{˘}{M}}_{1}$* (in short, *rescaled Belief–φ–divergence*) as (cf. [44])

(41) $$\begin{array}{ccc} D_{\varphi }\left({\overset{˘}{M}}_{2},{\overset{˘}{M}}_{1}\right) & := & D_{\varphi }\left(P^{{\overset{˘}{M}}_{2}},P^{{\overset{˘}{M}}_{1}}\right)=\sum_{k=1}^{2^{K}}p_{k}^{{\overset{˘}{M}}_{1}}\cdot \varphi \left(\frac{p_{k}^{{\overset{˘}{M}}_{2}}}{p_{k}^{{\overset{˘}{M}}_{1}}}\right)=\sum_{k=2}^{2^{K}}{\overset{˘}{M}}_{1}\left(A_{k}\right)\cdot \varphi \left(\frac{{\overset{˘}{M}}_{2}\left(A_{k}\right)}{{\overset{˘}{M}}_{1}\left(A_{k}\right)}\right)\\ & = & \sum_{k=2}^{2^{K}}\frac{M_{1}\left(A_{k}\right)}{2^{|A_{k}|}-1}\cdot \varphi \left(\frac{M_{2}\left(A_{k}\right)}{M_{1}\left(A_{k}\right)}\right)\;\geq 0\;, \end{array}$$

where for $A_{1}:=\emptyset$ we have used the convention that $0\cdot \varphi (\frac{0}{0}):=0$ (depending on φ, other zero rescaled belief masses may have to be excluded for finiteness). For the corresponding minimization problem

$$\begin{array}{ccc} & & \underset{{\overset{˘}{M}}_{2}\in \Omega^{resBBA}}{\inf}D_{\varphi }\left({\overset{˘}{M}}_{2},{\overset{˘}{M}}_{1}\right):=\underset{P^{{\overset{˘}{M}}_{2}}\in \Omega }{\inf}D_{\varphi }\left(P^{{\overset{˘}{M}}_{2}},P^{{\overset{˘}{M}}_{1}}\right) \end{array}$$

where $\Omega^{resBBA}$ is a collection of rescaled BBAs whose vectors of rescaled BBA values form the set Ω satisfying (2) and (4), we can apply Theorem 1 and (7) (with $2^{K}$ instead of *K*)—unless there is a more restrictive constraint that violates (2) such as, e.g., $\sum_{k=1}^{2^{K}}p_{k}^{{\overset{˘}{M}}_{2}}=A$, for which Theorem 2 (and thus, (17)) can be employed.

We can also apply our BS optimization method to “crossover cases” $D_{\varphi }\left(M_{2},P\right):=D_{\varphi }\left(P^{M_{2}},P\right)$ and $D_{\varphi }\left(P,M_{1}\right):=D_{\varphi }\left(P,P^{M_{1}}\right)$ (instead of (39)), $D_{\varphi }\left({\overset{˘}{M}}_{2},P\right):=D_{\varphi }\left(P^{{\overset{˘}{M}}_{2}},P\right)$ and $D_{\varphi }\left(P,{\overset{˘}{M}}_{1}\right):=D_{\varphi }\left(P,P^{{\overset{˘}{M}}_{1}}\right)$ (instead of (41)), $R_{\gamma }\left(M_{2},P\right):=R_{\gamma }\left(P^{M_{2}},P\right)$ and $R_{\gamma }\left(P,M_{1}\right):=R_{\gamma }\left(P,P^{M_{1}}\right)$, $R_{\gamma }^{nn}\left(M_{2},P\right):=R_{\gamma }^{nn}\left(P^{M_{2}},P\right)$ and $R_{\gamma }^{nn}\left(P,M_{1}\right):=R_{\gamma }^{nn}\left(P,P^{M_{1}}\right)$, $I\left(M_{2},P\right):=I\left(P^{M_{2}},P\right)$ and $I\left(P,M_{1}\right):=I\left(P,P^{M_{1}}\right)$, $\tilde{I}\left(M_{2},P\right):=\tilde{I}\left(P^{M_{2}},P\right)$ and $\tilde{I}\left(P,M_{1}\right):=\tilde{I}\left(P,P^{M_{1}}\right)$, $D_{\tilde{c}\cdot \varphi_{\gamma }}\left(M_{2},P\right):=D_{\tilde{c}\cdot \varphi_{\gamma }}\left(P^{M_{2}},P\right)$ and $D_{\tilde{c}\cdot \varphi_{\gamma }}\left(P,M_{1}\right):=D_{\tilde{c}\cdot \varphi_{\gamma }}\left(P,P^{M_{1}}\right)$, where $P\in R^{2^{K}}$ (respectively, $R_{\geq 0}^{2^{K}}$, $R_{>0}^{2^{K}}$, $A\cdot S_{\geq 0}^{2^{K}}$ or $A\cdot S_{>0}^{2^{K}}$) is a general vector (not necessarily induced by a (rescaled) BBA) having the same dimension (namely, $2^{K}$) as the (rescaled) BBA-induced vector to be compared with. For instance, if we apply Remark 2 to **P**:=${\mathbb{P}}^{unif}:=\left(\frac{1}{2^{K}},\ldots ,\frac{1}{2^{K}}\right)$ and $Q:=P^{M_{2}}$ and employ the corresponding straightforward application of the general results of Sections VIII and XII of [29], then we end up (via the appropriately applied Theorem 2) with the BS optimization results of $D_{\tilde{c}\cdot \varphi_{\gamma }}(M_{2},{\mathbb{P}}^{unif}$), $R_{\gamma }(M_{2},{\mathbb{P}}^{unif}$), $I(M_{2},{\mathbb{P}}^{unif}$) and $\tilde{I}(M_{2},{\mathbb{P}}^{unif}$) , which can be deterministically transformed into the BS optimization results of various different versions of entropies $E\left(M_{2}\right):=E\left(P^{M_{2}}\right)$ of BBAs $M_{2}$, where E(·) is any entropy in Chapter VIII of [29].

To give a more explicit example concerning the preceding paragraph, our BS method of Theorem 2 and (17) (with $2^{K}$ instead of *K* as well as $A=M_{P^{M_{1}}}=1$) applies to the crossover case

$$\begin{array}{ccc} & & \underset{M\in \Omega^{BBA}}{\inf}D_{\tilde{c}\cdot \varphi_{\gamma }}\left(M,P\right):=\underset{P^{M}\in \Omega }{\inf}D_{\tilde{c}\cdot \varphi_{\gamma }}\left(P^{M},P\right)\;\gamma \in R\setminus ]1,2\left[\;,\;\;\tilde{c}\in \;\right]0,\infty [\;, \end{array}$$

where $P^{M}$ is a vector of *M*–based BBA values and $\Omega^{BBA}$ is as above, and P is a vector whose sum of components may not necessarily be one. For instance, for the special choice γ=1, i.e., $\varphi \left(t\right):=\varphi_{1}\left(t\right):=t\cdot \log t+1-t\in [0,\infty [$ (cf. (8)), $P^{M}:={\left(p_{k}^{M}\right)}_{k=1,\ldots ,2^{K}}:={\left(M(A_{k})\right)}_{k=1,\ldots ,2^{K}}$, $P:={\left(p_{k}\right)}_{k=1,\ldots ,2^{K}}$ with $p_{k}:=2^{|A_{k}|}-1$ employing the cardinality $|A_{k}|$ of $A_{k}$, and the usual convention $0\cdot \log(\frac{0}{0}):=0$, we end up with (cf. (9))

$$D_{\varphi_{1}}\left(M,P\right)=D_{\varphi_{1}}\left(P^{M},P\right)=\sum_{k=2}^{2^{K}}M\left(A_{k}\right)\cdot \log\left(\frac{M(A_{k})}{2^{|A_{k}|}-1}\right)-1+\sum_{k=2}^{2^{K}}\left(2^{|A_{k}|}-1\right)=:-E^{DE}\left(M\right)-1+\sum_{k=2}^{2^{K}}\left(2^{|A_{k}|}-1\right)$$

where $E^{DE}\left(M\right):=-\sum_{k=2}^{2^{K}}M\left(A_{k}\right)\cdot \log\left(\frac{M(A_{k})}{2^{|A_{k}|}-1}\right)\geq 0$ is nothing but (a multiple of) Deng’s entropy of the BBA *M* (cf. [50], see also, e.g., [51]). Of course, we can also deal with the optimization of corresponding Renyi divergences $\inf_{M\in \Omega^{BBA}}R_{\gamma }\left(M,P\right)=\inf_{P^{M}\in \Omega }R_{\gamma }\left(P^{M},P\right)$ by applying (18) and (19). For the “reverse-argument” crossover case $\inf_{M\in \Omega^{BBA}}D_{\varphi }\left(P,M\right):=\inf_{P\in \Omega }D_{\varphi }\left(P,P^{M}\right)$ one can apply Theorem 1 (and thus, (7))—unless there is a more restrictive constraint, which violates (2) such as, e.g., $\sum_{k=1}^{2^{K}}p_{k}=A$, for which Theorem 2 (and hence, (17))—as well as its consequences (19), (21), (23), (24) (and thus, (25)–(28)) can be employed.

## 6. Conclusions

In this paper, we have—in terms of *generalized φ–divergences*—quantified the dissimilarity between fuzzy sets and some of their subsetups such as ν–rung orthopair fuzzy sets and extended representation type ν–rung orthopair fuzzy sets; crossover-cases of *generalized φ–divergences* between fuzzy set types and general vectors have been treated, too. Moreover, we have presented how to tackle corresponding constrained minimization problems by appropriately applying our recently developed dimension-free *bare (pure) simulation method* of [29]. Afterwards, an analogous program is carried out by defining *generalized φ–divergences* between (rescaled) basic belief assignments as well as between (rescaled) basic belief assignments and vectors, and tackling corresponding constrained minimization problems.

As an outlook for future work, it is interesting to study ways of efficiently simulating the corresponding minimizers (in addition to the here-addressed minima). This ongoing research will appear in a future paper. 

## Data Availability

No new data were created or analyzed in this study. Data sharing is not applicable to this article.

## References

[1] Csiszár I. (1963). Eine informationstheoretische Ungleichung und ihre Anwendung auf den Beweis der Ergodizität von Markoffschen Ketten. Publ. Math. Inst. Hungar. Acad. Sci..

[2] Ali M.S., Silvey D. (1966). A general class of coefficients of divergence of one distribution from another. J. Roy. Statist. Soc. B.

[3] Morimoto T. (1963). Markov processes and the H-theorem. J. Phys. Soc. Jpn..

[4] Liese F., Vajda I. (1987). Convex Statistical Distances.

[5] Read T.R.C., Cressie N.A.C. (1988). Goodness-of-Fit Statistics for Discrete Multivariate Data.

[6] Vajda I. (1989). Theory of Statistical Inference and Information.

[7] Csiszár I., Shields P.C. (2004). Information Theory and Statistics: A Tutorial.

[8] Pardo L. (2006). Statistical Inference Based on Divergence Measures.

[9] Liese F., Miescke K.J. (2008). Statistical Decision Theory: Estimation, Testing, and Selection.

[10] Liese F., Vajda I. (2006). On divergences and informations in statistics and information theory. IEEE Trans. Inf. Theory.

[11] Vajda I., van der Meulen E.C., Karian Z.A., Dudewicz E.J. (2010). Goodness-of-fit criteria based on observations quantized by hypothetical and empirical percentiles. Handbook of Fitting Statistical Distributions with R.

[12] Reid M.D., Williamson R.C. (2011). Information, divergence and risk for binary experiments. J. Mach. Learn. Res..

[13] Basseville M. (2013). Divergence measures for statistical data processing—An annotated bibliography. Signal Process..

[14] Lindsay B.G., Taper M.P., Lele S.R. (2004). Statistical distances as loss functions in assessing model adequacy. The Nature of Scientific Evidence.

[15] Lindsay B.G., Markatou M., Ray S., Yang K., Chen S.-C. (2008). Quadratic distances on probabilities: A unified foundation. Ann. Statist..

[16] Markatou M., Sofikitou E. (2018). Non-quadratic distances in model assessment. Entropy.

[17] Markatou M., Chen Y. (2019). Statistical distances and the construction of evidence functions for model adequacy. Front. Ecol. Evol..

[18] Broniatowski M., Stummer W., Nielsen F., Rao A.S.R.S., Rao C.R. (2022). A unifying framework for some directed distances in statistics. Geometry and Statistics.

[19] Zadeh L.A. (1965). Fuzzy sets. Inf. Control.

[20] Dempster A.P. (1967). Upper and lower probabilities induced by a multivalued mapping. Ann. Math. Stat..

[21] Shafer G. (1976). A Mathematical Theory of Evidence.

[22] Bhandari D., Pal N.R. (1993). Some new information measures for fuzzy sets. Inf. Sci..

[23] Vlachos I.K., Sergiadis G.D. (2007). Intuitionistic fuzzy information—Applications to pattern recognition. Pattern Recogn. Lett..

[24] Xiao F., Ding W. (2019). Divergence measure of Pythagorean fuzzy sets and its application in medical diagnosis. Appl. Soft. Comput. J..

[25] Xiao F. (2019). Multi-sensor data fusion based on the belief divergence measure of evidences and the belief entropy. Inf. Fusion.

[26] Xiao F. (2020). A new divergence measure for belief functions in D-S evidence theory for multisensor data fusion. Inf. Sci..

[27] Li J., Xie B., Jin Y., Hu Z., Zhou L. (2020). Weighted conflict evidence combination method based on hellinger distance and the belief entropy. IEEE Access.

[28] Yager R.R. (2017). Generalized orthopair fuzzy sets. IEEE Trans. Fuzzy Syst..

[29] Broniatowski M., Stummer W. (2023). A precise bare simulation approach to the minimization of some distances. I. Foundations. IEEE Trans. Inf. Theory.

[30] Stummer W., Vajda I. (2010). On divergences of finite measures and their applicability in statistics and information theory. Statistics.

[31] Broniatowski M., Keziou A. (2006). Minimization of *ϕ*-divergences on sets of signed measures. Stud. Scient. Math. Hungar..

[32] Broniatowski M., Stummer W., Nielsen F. (2019). Some universal insights on divergences for statistics, machine learning and artificial intelligence. Geometric Structures of Information.

[33] Csiszár I. (1984). Sanov property, generalized I-projection and a conditional limit theorem. Ann. Probab..

[34] Broniatowski M., Stummer W., Nielsen F., Barbaresco F. (2023). On a cornerstone condition of bare-simulation distance/divergence optimization. Geometric Science of Information GSI 2023.

[35] Burbea C., Rao C.R. (1982). On the convexity of some divergence measures based on entropy functions. IEEE Trans. Inf. Theory.

[36] Lin J. (1991). Divergence measures based on the Shannon entropy. IEEE Trans. Inf. Theory.

[37] Pardo M.C., Vajda I. (1997). About distances of discrete distributions satisfying the data processing theorem of information theory. IEEE Trans. Inf. Theory.

[38] Topsoe F. (2000). Some inequalities for information divergence and related measures of discrimination. IEEE Trans. Inf. Theory.

[39] Endres D.M., Schindelin J.E. (1993). A new metric for probability distributions. IEEE Trans. Inf. Theory.

[40] Vajda I. (2009). On metric divergences of probability measures. Kybernetika.

[41] Sason I. Tight bounds for symmetric divergence measures and a new inequality relating *f*-divergences. Proceedings of the 2015 IEEE Information Theory Workshop (ITW).

[42] Renyi A., Neyman J. (1961). On measures of entropy and information. Proceedings of the Fourth Berkeley Symposium on Mathematical Statistics and Probability.

[43] van Erven T., Harremoes P. (2014). Renyi divergence and Kullback–Leibler divergence. IEEE Trans. Inf. Theory.

[44] Broniatowski M., Stummer W. (2021). A precise bare simulation approach to the minimization of some distances. Foundations. arXiv.

[45] Atanassov K.T. (1986). Intuitionistic fuzzy sets. Fuzzy Sets Syst..

[46] Yager R.R. Pythagorean fuzzy subsets. Proceedings of the 2013 Joint IFSA World Congress and NAFIPS Annual Meeting (IFSA/NAFIPS).

[47] Yager R.R., Abbasov A.M. (2013). Pythagorean membership grades, complex numbers, and decision making. Int. J. Intell. Syst..

[48] Verma R. (2020). Multiple attribute group decision-making based on order-*α* divergence and entropy measures under q-rung orthopair fuzzy environment. Int. J. Intell. Syst..

[49] Huang J., Song X., Xiao F., Cao Z., Lin C.-T. (2023). Belief *f*–divergence for EEG complexity evaluation. Inf. Sci..

[50] Deng Y. (2016). Deng entropy. Chaos Solitons Fract..

[51] Kang B., Deng Y. (2020). The maximum Deng entropy. IEEE Access.

